# Transforming growth factor β plays an important role in enhancing wound healing by topical application of Povidone-iodine

**DOI:** 10.1038/s41598-017-01116-5

**Published:** 2017-04-20

**Authors:** Li Wang, Wenhan Qin, Yaying Zhou, Bin Chen, Xiaoqing Zhao, Hailin Zhao, Emma Mi, Ella Mi, Qingmei Wang, Jiaolin Ning

**Affiliations:** 1 Nursing department of Operation theater, Southwest Hospital, Third Military Medical University, 29 Gaotanyan Road, Chongqing, 400038 China; 2 Department of Anesthesiology, Southwest Hospital, Third Military Medical University, 29 Gaotanyan Road, Chongqing, 400038 China; 3 Nursing Department, General hospital of the Chinese People’s Liberation Army, 28 Fuxing Road, Beijing, 100853 China; 4 Anaesthetics, Pain Medicine and Intensive Care, Department of Surgery and Cancer, Faculty of Medicine, Imperial College London, Chelsea & Westminster Campus, London, United Kingdom

## Abstract

Povidone-iodine (PVI) is principally used as an antimicrobial agent. It has been found that 0.5% PVI can attenuate congestion, edema and pain induced by pressure sores. Thus this study aimed to assess the effects of 0.5% PVI on acute skin wounds. Four full-thickness excisional wounds were generated on the dorsal skin of male Sprague-Dawley rats with a 10-mm sterile punch. Two wounds were left untreated and the other two were dressed with gauze with 0.5% PVI for 1 hour per day for the first 5 days after injury. 10-mm full-thickness excisional wounds were also generated on the dorsal skin of rats treated with 10 mg/kg SB431542 and all wounds were treated with 0.5% PVI for 5 days. PVI treatment enhanced wound healing via promotion of expression of α SMA and TGF β, neovascularization and re-epithelialization. Interleukin 6 was reduced following PVI treatment. Inhibition of TGF β abolished the effect of PVI treatment on wound closure. These data show that topical application of 0.5% PVI could promote acute skin wound healing though increased expression of TGF β leading to enhanced formation of granulation tissue, even in the absence of obvious infection.

## Introduction

Local treatments for skin wounds aim to prevent infection and enhance healing of the wound. Ideal agents have both anti-microbial and wound healing-promoting properties, as well as potential for easy and extensive topical application in clinical settings. Povidone-iodine (PVI) disinfectant is the most commonly used topical agent for sterilization of skin, including in surgical settings^1, 2^. Various disinfectants constituted with iodine have been reported to produce effects on skin wound healing via multiple mechanisms, including prevention of bacterial growth, acceleration of granulation tissue formation^3–7^, e.t.c. Recently, Kanno *et al*. demonstrated that 1% polyvinylpyrrolidone-iodine could promote healing of contaminated wounds as it reduces wound bacterial counts and enhances re-epithelialization^8, 9^. Several mixed products containing iodine, such as Repithel (PVP-ILH), a new liposomal hydrogel formulation with polyvinylpyrrolidone-iodine, have also been reported to prevent infection and promote healing of chronic, burn, infected or contaminated wounds, although the content of iodine in the mixed formulation is often higher than that of iodine-containing disinfectants in clinical use^10, 11^. We found that 0.5% PVI disinfectant could attenuate congestion, edema and pain induced by pressure sores. However the effects of 0.5% PVI disinfectant on acute skin wound healing remains unknown. Therefore, in this study full thickness skin wounds were created to evaluate the effect of 0.5% PVI on the wound healing process and its underlying mechanisms in rats.

## Results

### PVI treatment enhanced wound closure and promoted granulation tissue formation and maturation

**Figure 1:**
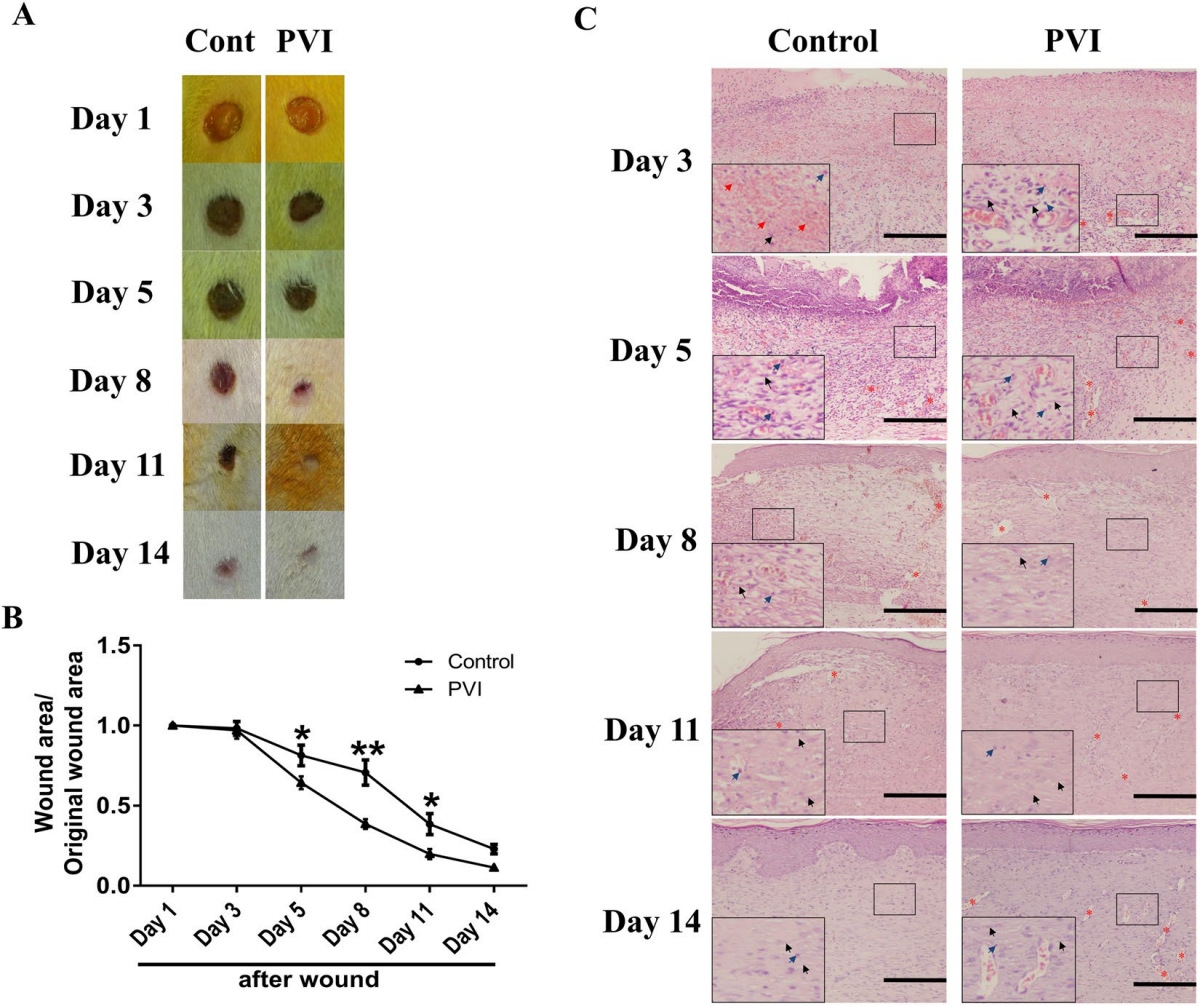
Povidone-iodine (PVI) treatment enhanced cutaneous wound closure and granulation tissue formation and maturation. (**A**) Representative photographs of wounds from a control and PVI treated rat. (**B**) Wound contraction (%) at day 1 to 14 post-injury. (**C**) Representative micrographs of H&E stained wound sections. Red arrows indicate red blood cells. Blue arrows indicate inflammatory cells. Grey arrows indicate fibroblasts. Red asterisks indicate new blood vessels. Scale bar = 50 μm. *p < 0.05, **p < 0.01 *vs* control. Data are means ± S.D, n = 12.

Pictures of skin wounds from control and PVI-treated rats are shown in Fig. 1A. The excisional wound healing rates in both groups are shown in Fig. 1B. The healing curves (Fig. 1B) demonstrate that there was a rapid acceleration of healing in the wounds treated with PVI at day 5, 8 and 11 post-injury (p < 0.05). At day 3 post-injury, the wound field showed abundant red blood cells and some inflammatory cell infiltration. At day 5, thin immature granulation tissue, dominated by inflammatory cells and new blood vessels and with few fibroblasts and collagen deposition, was present in the skin wound area. At day 8 and 11, the healing wound consisted of moderately thick granulation tissue, with increased numbers of fibroblasts and collagen deposition, more neovascularization, minimal moderate epithelial layer formation and few inflammatory cells in the epithelial layer can be seen in wound area. Day 14 after injury this progressed to thick mature granulation tissue characterised by compact collagen parallel to the well-formed complete epithelial layer and decreased numbers of fibroblasts and new blood vessels. All these changes were more pronounced in PVI-treated wounds than in control wounds (Fig. 1C).

### PVI treatment promoted alpha smooth muscle actin (α-SMA) expression in wounds

**Figure 2:**
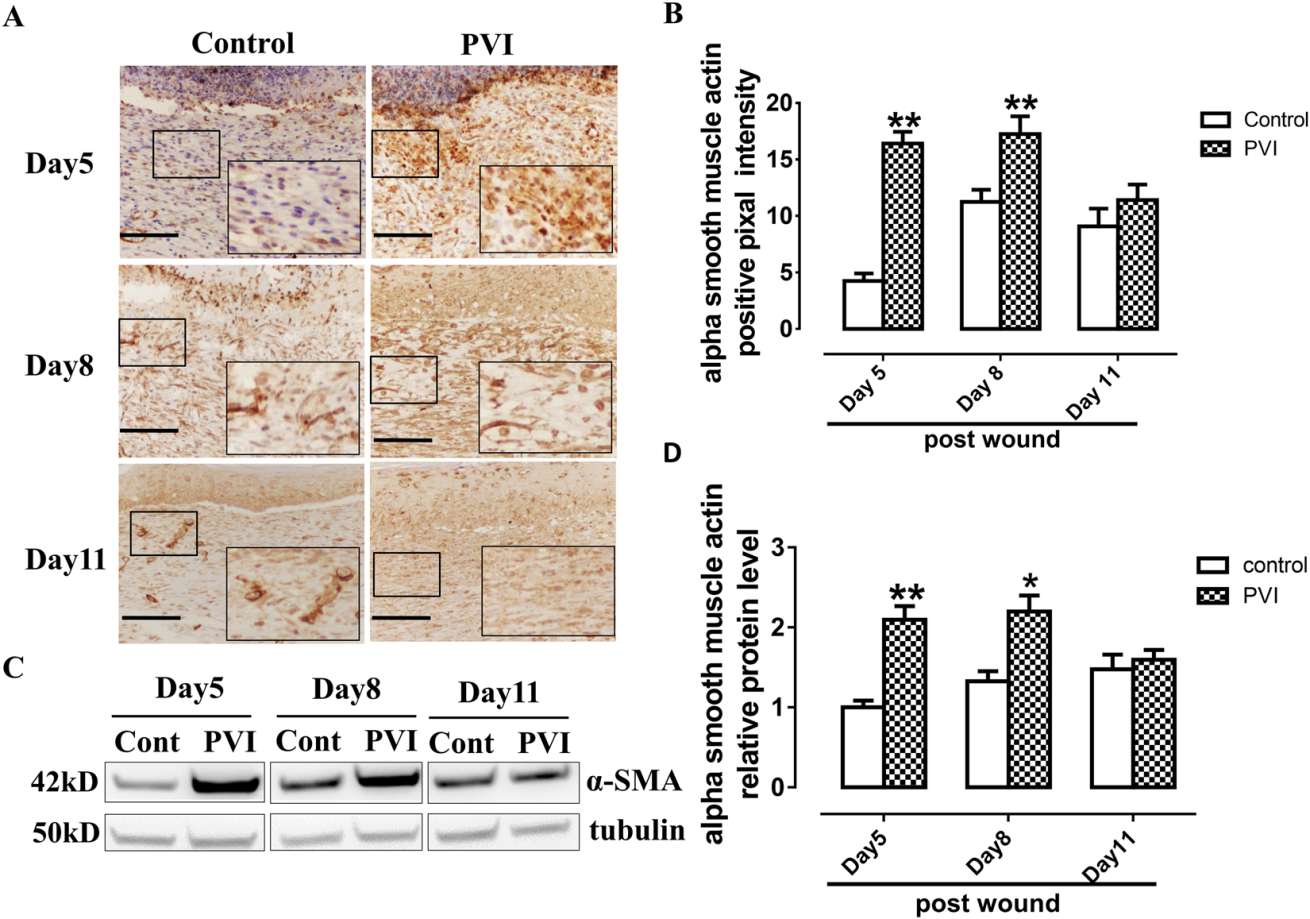
Povidone-iodine (PVI) treatment promoted alpha smooth muscle actin (α-SMA) expression. (**A**) Representative immunohistochemical α-SMA stained wound sections from a control and PVI treated rat. Scale bar = 50 μm. (**B**) Semi-quantitative analysis of α-SMA expression. (**C**) Representative western blotting for α-SMA in wound areas. (**D**) Semi-quantitative analysis of α-SMA expression, n = 4. *p < 0.05, **p < 0.01 *vs* control. Data are means ± S.D, n = 24.

α-SMA is a known marker of myofibroblast cells in the skin, which play important roles in the closure of cutaneous wounds. As shown in Fig. 2A, α-SMA expression peaked at the day 8 after injury. PVI treatment enhanced α-SMA expression at days 5 and 8 compared to controls as shown by immunohistochemistry (p < 0.01) (Fig. 2B), which was confirmed by analysis of α-SMA expression with western blotting (Fig. 2C,D).

### PVI treatment enhanced neovascularization in wounds

**Figure 3:**
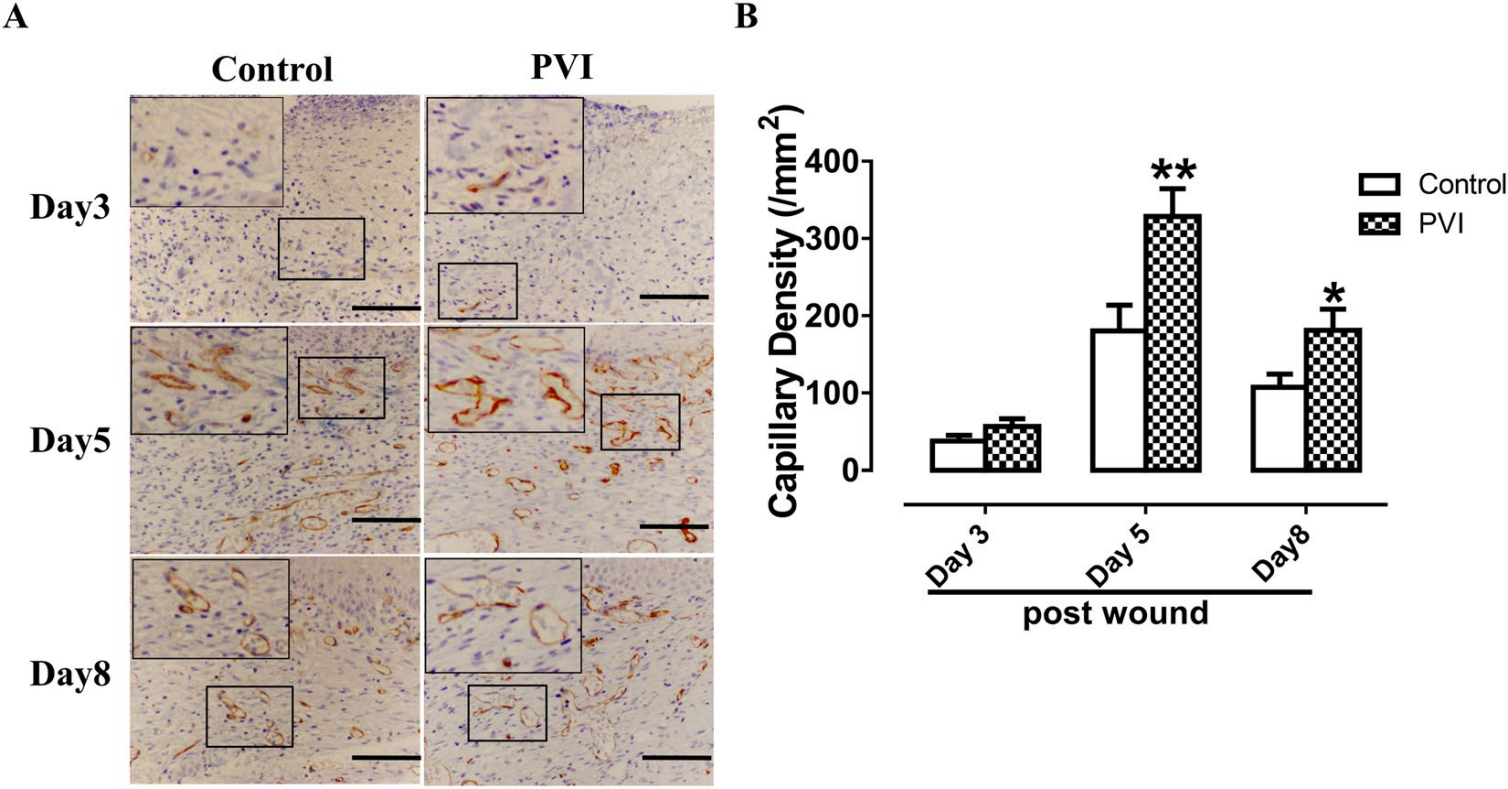
Povidone-iodine (PVI) treatment promoted wound neovascularization. (**A**) Representative immunohistochemical CD34 stained wound sections from a control and PVI treated rat. Scale bar = 50 μm. (**B**) Semi-quantitative analysis of capillary density (/mm^2^). Capillary density was assessed by counting the number of CD34 positive microvessels in high-power fields (40x). *p < 0.05, **p < 0.01 *vs* control. Data are means ± S.D, n = 24.

CD34 is a marker of endothelial progenitor cells (EPCs). CD34+ cells appeared in the wound areas at day 3 post-injury, and peaked at day 5, then gradually decreasing over time (Fig. 3A), CD34+ cells were almost nonexistent in the skin wound area at day 11 and 14 (data not shown here). PVI treated wounds showed a significant increase in CD34+ cells when compared with controls at day 5 (p < 0.01) and day 8 (p < 0.05) post-injury (Fig. 3B).

### PVI treatment promoted transforming growth factor (TGF) β expression

**Figure 4:**
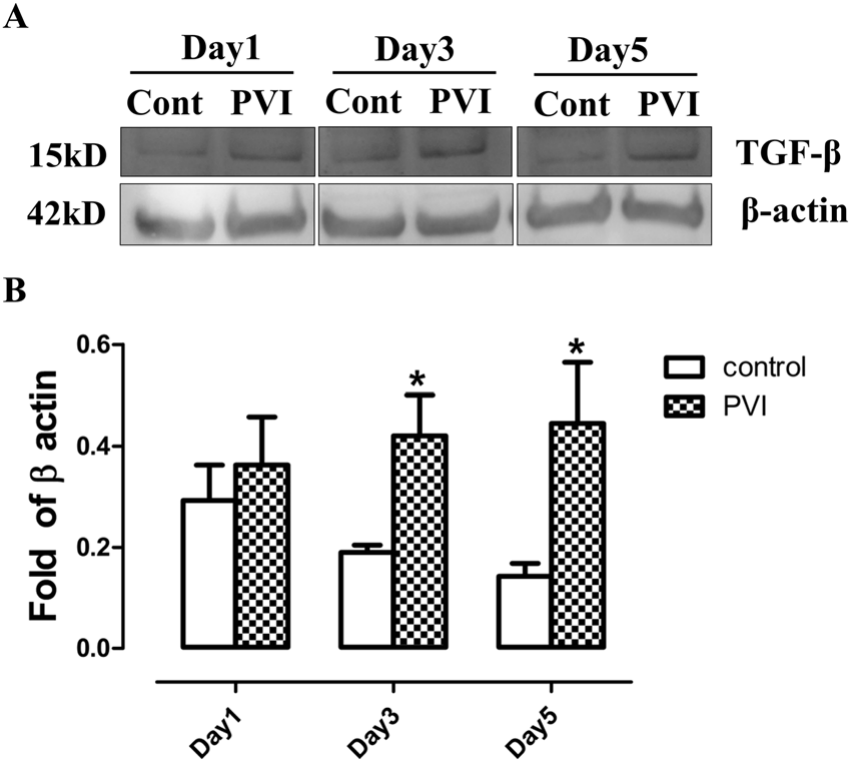
Povidone-iodine (PVI) treatment increased transforming growth factor β expression. (**A**) Representative western blotting for transforming growth factor (TGF) β in wound areas. (**B**) Semi-quantitative analysis of TGF-β expression. *p < 0.05 *vs* control. Data are means ± S.D, n = 4.

TGF-β is released in wound areas following tissue damage, through platelet degranulation which plays a key role in the process of wound healing. TGF-β was detected 1 day after injury, gradually increased over time, and was higher in wounds treated with PVI than in control wounds (Fig. 4A,B).

### The effects of PVI treatment on macrophage infiltration/accumulation into wound areas

**Figure 5:**
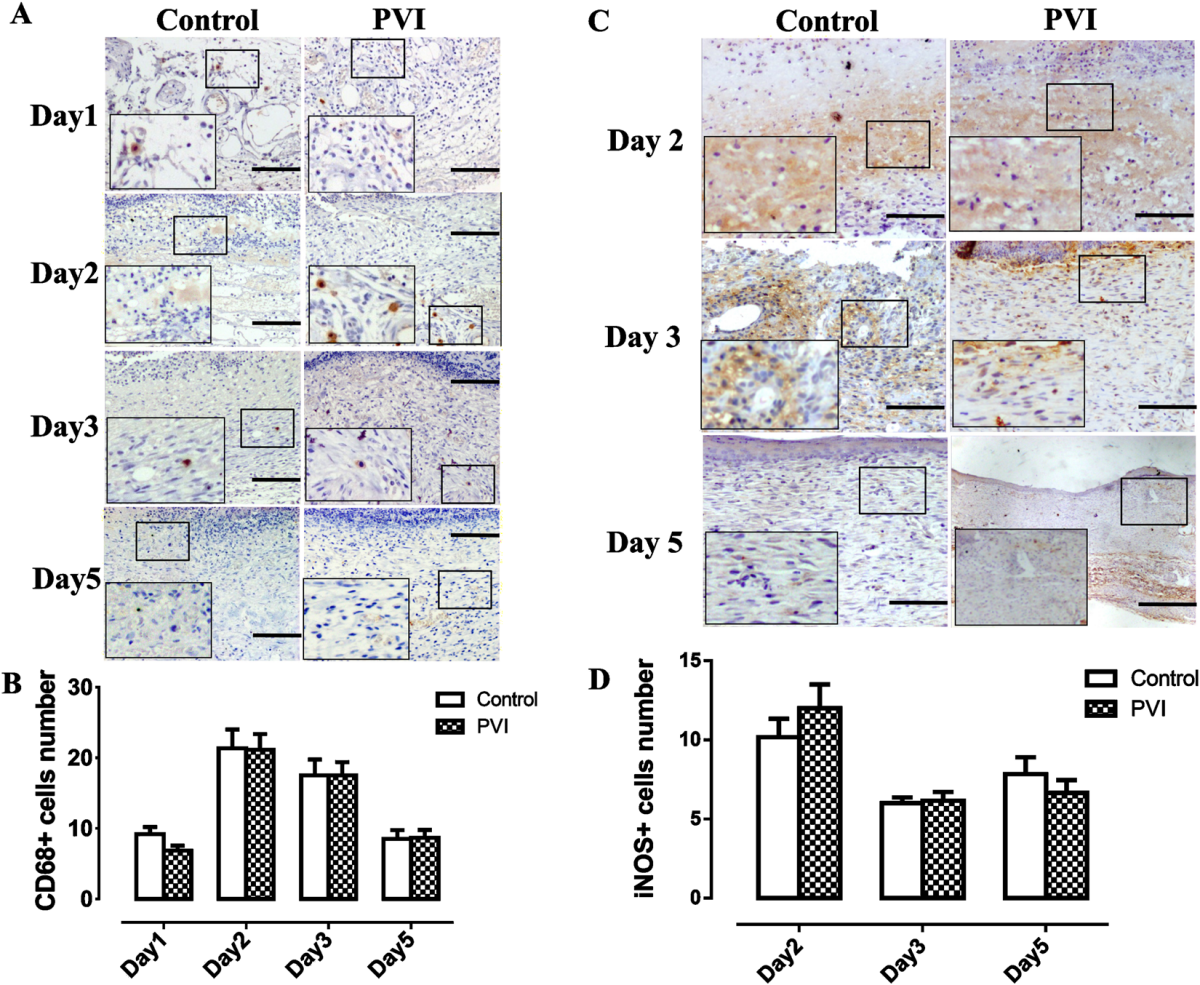
The effect of povidone-iodine (PVI) treatment on macrophages. (**A**) Representative immunohistochemical CD68 stained wound sections. Scale bar = 50 μm. (**B**) Quantitative analysis of CD68+ cells (/high power fields). (**C**) Representative immunohistochemical inducible nitric oxide synthase (iNOS) stained wound sections. Scale bar = 50 μm. (**D**) Quantitative analysis of iNOS + cells (/high power fields). Data are means ± S.D, n = 24.

The inflammatory phase, when cells, including neutrophils and macrophages, are recruited to the wound site, is the first phase of wound healing. Mainly derived from bloodstream monocytes and resident macrophages, macrophages in wound areas can be detected using CD68+ as a marker. As shown by Fig. 5A, macrophages were found at day 1 post-injury, peaked at day 2, and decreased over time after day 3. The numbers of CD68+ cells in PVI treated wounds were similar to that in control wounds at day 1, 2, 3 and 5 post-injury (Fig. 5B).

Induced nitric oxide synthase (iNOS) was used as marker of M1 macrophages. Numbers of iNOS+ cells in PVI treated wound areas were not lower than that in control wound areas at 2^nd^, 3^rd^ and 5^th^ days post-injury (p > 0.05) (Fig. 5C,D).

### The effects of PVI treatment on the tumor necrosis factor α and interleukin 6 release in the wound areas

**Figure 6:**
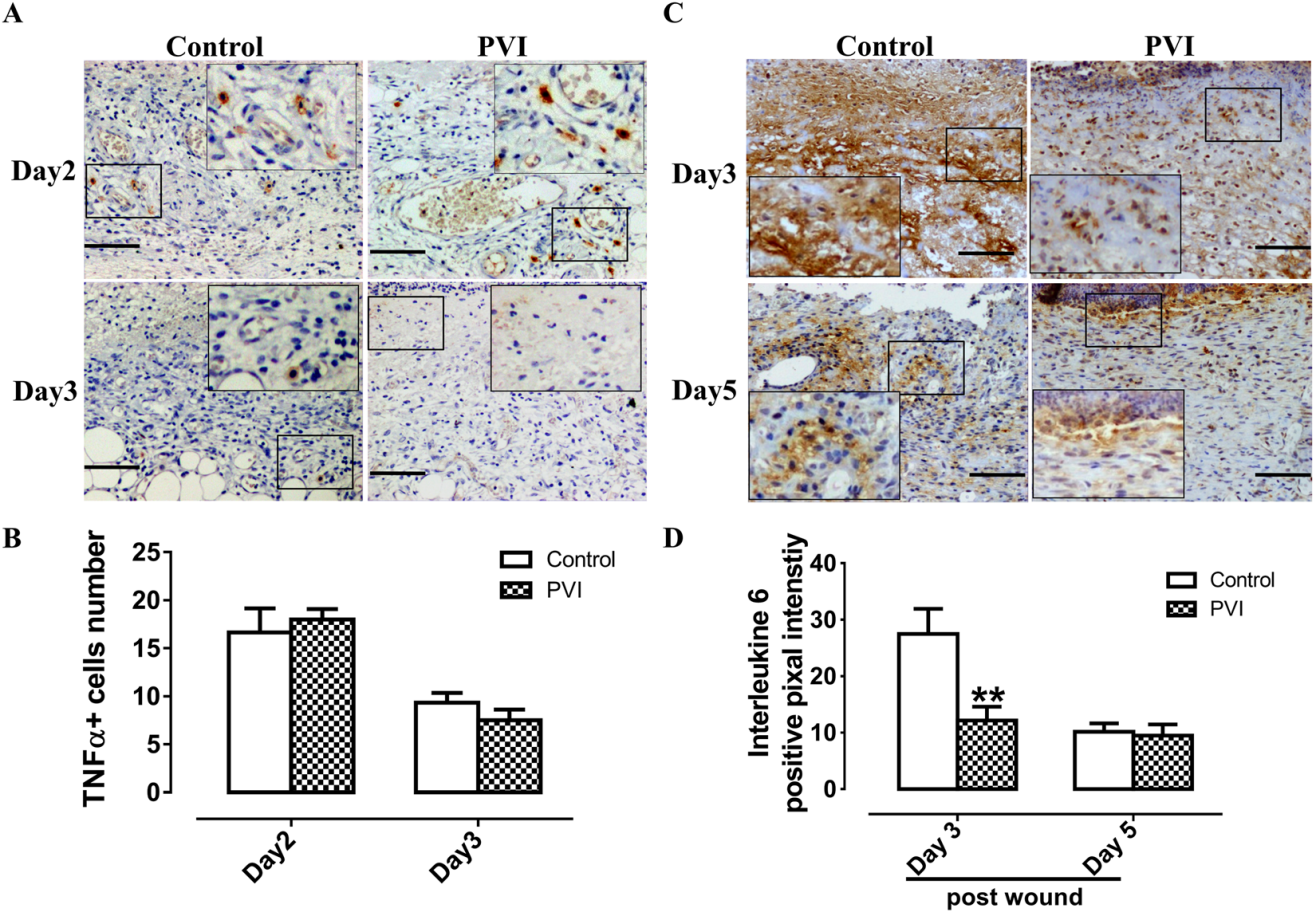
The effect of povidone-iodine (PVI) treatment on tumor necrosis factor (TNF) α and interleukin (IL) -6 expression at the wound site. (**A**) Representative immunohistochemical TNFα stained wound sections. Scale bar = 50 μm. (**B**) Semi-quantitative analysis of TNFα + cells (/high power fields). (**C**) Representative immunohistochemical IL-6 stained wound sections. Scale bar = 50 μm. (**D**) Semi-quantitative analysis of IL-6 expression. **p < 0.01 *vs* control. Data are means ± S.D, n = 24.

Tumor necrosis factor (TNF) α is produced early in wounds after insult, mainly by M1 macrophages. TNFα was largely located in the cytoplasm of cells (Fig. 6A). The numbers of TNFα+ cells in PVI treated wounds did not differ from that in control wounds as shown in Fig. 6B (p > 0.05).

Inflammatory cells including neutrophils and monocytes infiltrate into the wound and release cytokines which participate in the healing process. IL-6 was not detected in the wound area at day 1 post-injury (data not shown), but peaked at day 3 (Fig. 6C), and then gradually decreased, such that it was not present in the wound area at day 8 and 11 post-injury (data not shown). Animals treated with PVI exhibited a significant decrease in wound IL-6 level compared to control animals at day 3 post-injury (p < 0.01) (Fig. 6D).

### PVI treatment promotes differentiation of epithelial cells

**Figure 7:**
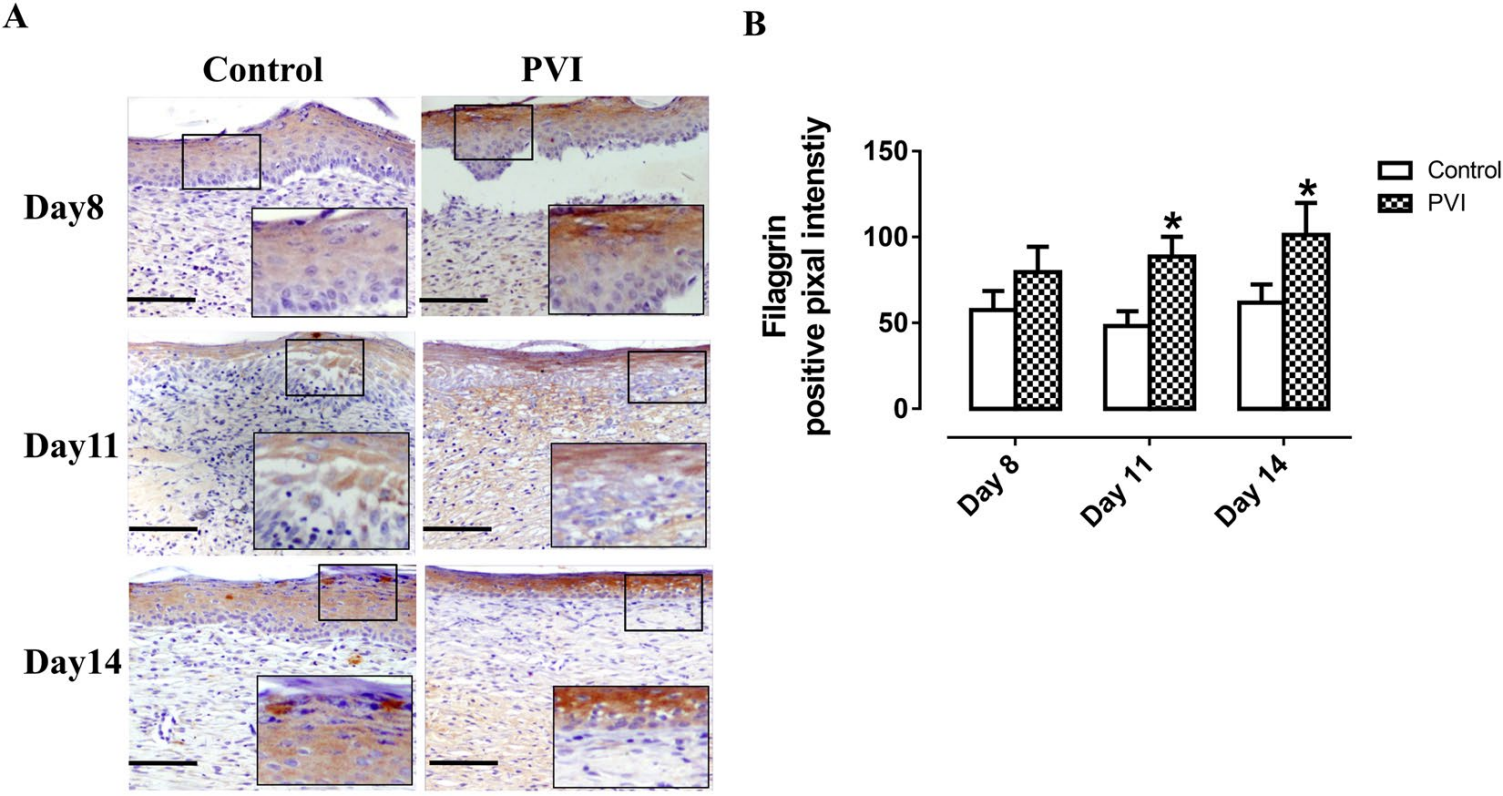
Povidone-iodine (PVI) treatment enhanced filaggrin expression. (**A**) Representative immunohistochemical filaggrin stained wound sections. Scale bar = 50 μm. (**B**) Semi-quantitative analysis of filaggrin expression. *p < 0.05 *vs* control. Data are means ± S.D, n = 24.

Expression of filaggrin, a marker of epithelial cell differentiation, increased over time after injury (Fig. 7A). Filaggrin was detected in the wound area at day 8. PVI treatment increased filaggrin expression compared to control at day 11 and 14 post-injury (p < 0.05) (Fig. 7B).

### Inhibition of TGF-β reversed the wound healing process enhanced by PVI treatment

**Figure 8:**
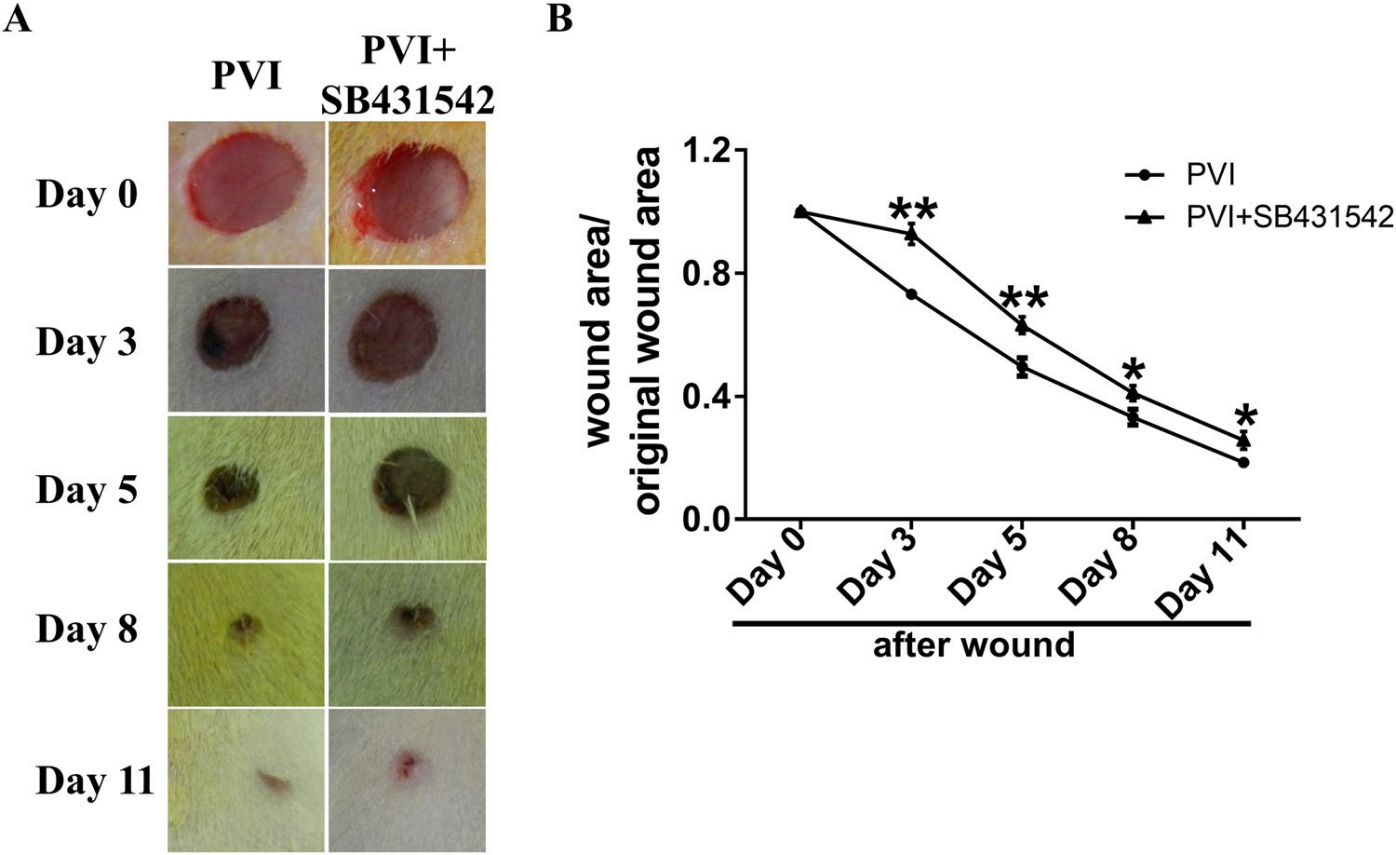
Inhibition of transforming growth factor β prevented the promotion of skin wound healing by Povidone-iodine (PVI) treatment. (**A**) Representative photographs of wounds from rats treated with inhibitor of transform growth factor β (SB431542) or vehicle and PVI. (**B**) Wound contraction (%) at day 1 to 11 post-injury. *p < 0.05, **p < 0.01 *vs* control. Data are means ± S.D, n = 12.

SB431542 is a small molecule inhibitor of TGF-β. SB431542 treatment delayed the closure of skin wounds in rats treated with PVI (Fig. 8A). The healing curve indicates that inhibition of TGF-β significantly delayed the wound healing process after PVI treatment (Fig. 8B).

## Discussion

Our data showed that topical application of 0.5% PVI promotes acute cutaneous wound healing, and that TGF-β has an important role in this process. The healing process of cutaneous wounds involves numerous cell types, including neutrophils, macrophages, fibroblasts and endothelial cells. TGF-β plays a central role in every phase of wound healing^12–15^; in general, it suppresses the inflammatory response and promotes the formation of granulation tissue in wounded areas^12^. In the current study, it was found that PVI treatment promoted wound closure and granulation tissue formation, and moreover, granulation tissue was more organized. Increased myofibroblast activity contributes to wound closure^16, 17^. The increased expression of α-SMA in PVI treated wounds suggests that PVI treatment may promote phenotype switch of fibroblast to myofibroblast via increased expression of TGF-β. This was confirmed by the result which inhibition of TGF-β with SB431542 abolished the positive effects of PVI treatment on wound closure.

Neovascularisation initiates formation of granulation tissue^18^. Thus, organized formation of granulation tissue following PVI treatment may be attributed to the increased growth of new vessels. TGF-β stimulates endothelial cell migration and angiogenesis, so it is probable that PVI treatment augmented neovascularization at least partially through up-regulation of TGF-β.

TGF-β inhibits keratinocyte proliferation and has inhibitory effects on re-epithelialization in the skin healing process^13^. However, re-epthelialization was not suppressed by PVI induced upregulation of TGF-β expression, implying that other mechanisms which promote re-epthelialization may be involved in the promotion of wound healing by PVI. Further studies are warranted to clarify this.

Filaggrin is a crucial for wound healing and aggregates the keratin filaments into tight bundles, promoting the collapse of the cell into a flattened shape^19, 20^. Increased levels of filaggrin might not indicate that TGF-β enhances its levels directly. TGB-β might promote differentiation of epithelial cell and consequently increase of filaggrin^19^. Further studies are warranted to clarify their roles.

Interestingly, PVI treatment did not markedly influence the inflammatory phase of skin wound healing, as demonstrated by its lack of effects on the number and phenotype of macrophages and TNFα+ cells. However the level of IL-6 in wound areas in the early stages of wound healing was reduced by PVI treatment. IL-6 is a cytokine with both pro- and anti- inflammatory properties and is a marker of stress, with the ability to induce expression of acute phase proteins^21^. This suggests that PVI treatment may promote skin wound healing by attenuating the acute inflammatory response which involves congestion and edema, in line with the previous study.

The underlying mechanism of enhanced TGF-β level in the wounded skin by PVI treatment still remains unknown, it is uncertain whether PVI cause increase in its production through increase in mRNA levels or promote activation of TGF-β, in addition, which cell type plays the major role during PVI treatment remained to be clarified. All these needed to be explored in future studies.

In summary, our data showed that PVI enhanced skin wound healing though increased expression of TGF-β, increased neovascularization, and phenotype switch of fibroblast to myofibroblast. Re-epthelialization may be also involved in the process. Our study supports the clinical use of topical application of 0.5% PVI in the treatment of skin wounds without infection.

## Methods

### Wound model and PVI treatment

The study was approved by the research Ethics Committee of the Third Military Medical University, Chongqing, China, and all experiments were performed according to the guidelines regarding Care and Use of Laboratory Animals. Male Sprague-Dawley rats (250–260 g) (n = 12 per group) were obtained from the Third Military Medical University (Chongqing, China) and housed under 12 hours of light-dark conditions with free access to water and standard laboratory chow. After 12 hours fasting, rats were anesthetized with 2% Isoflurane and 80 mg/kg ketamine I.M.

Following this, their dorsal skin was shaved and sanitized with 70% ethanol, and 4 full-thickness excisional wounds were generated with a 10-mm sterile punch (Stiefel laboratories, Carolina, USA). Two wounds were left untreated while the other two were dressed with gauze with 0.5% PVI for 1 hour once a day from day 0 (the wounded day) to day 5.

Rats in another group underwent intraperitoneal (IP) injections of 10 mg/kg SB431542 (an inhibitor of transforming growth factor β, TGF-β) in 5% DMSO solution (vehicle) daily for 5 days. 4 full-thickness skin wounds were created on the backs of these rats using the methods described. These animals were housed individually after recovery from anesthesia. Wound area was regularly monitored with planimetric measurement, in which photographs of each wound were taken at indicated time points and then analyzed by Image J (NIH) (Image J, 1.47v, NIH, Bethesda, USA) to calculate the unhealed wound size.

### Histology

Wounded skin specimens were harvested from rat cohorts at day 3, 5, 8, 11 and 14 post-injury, fixed in 4% formaldehyde buffered with PBS (pH 7.2), and embedded in paraffin. These were sectioned into 5 μm sections and stained with H&E.

### Immunohistochemistry

Wounded skin specimens were harvested from rat cohorts at the same indicated days, fixed in 4% paraformaldehyde for 30 minutes, and subjected to cryosection. 5 μm cryosections were subjected to immunohistochemistry with primary antibody against alpha smooth muscle actin (α-SMA), CD34, CD68, inducible nitric oxide synthase (iNOS), TNFα, IL-6 and filaggrin (Abcam, Cambridge, UK). After washing, samples were incubated with biotinylated secondary antibodies, followed by incubation with avidin-HRP solution. Samples were developed with DAB solution (0.06% DAB in 0.05 M Tris, pH 7.6 with 0.03% H_2_O_2_).

Microphotographs from twenty random fields were captured (40x or 20x or 10x) for the semi-quantitative analysis of protein expression in the wound area. The number of positive staining cells was counted per area.

### Western blotting

The tissue was harvested at indicated time points and lysed in a lysis buffer (0.5% Nonidet P-40, 10 mM Tris, pH 7.4, 150 mM Nacl, 1 mM EDTA, 1 mM Na_3_VO_4_) with a protease inhibitor (1 mM PMSF). BCA assay was used to quantify protein level. Cell lysates (30 μg protein) were resolved by 10% sodium dodecyl sulfate-polyacrylamide gel electrophoresis (SDS-PAGE) (Immobilon; Millipore, Bedford, MA). For immunoblotting, primary antibodies against α-SMA, TGF-β, α-tubulin and β-actin (1:1000) (Abcam, Cambridge, UK) and IgG-HRP secondary antibody (1:2000) were used. Chemiluminsescent signals were acquired using Storm 860 PhosphorImager (Molecular Dynamics, Sunnyvale, CA). Densitometric analysis was performed using Image J 5.0 software. Four biological replicates were performed.

### Statistical Analysis

All statistical data are presented as mean ± SD. Data comparisons were performed by independent t tests (GraphPad Software, San Diego, CA). P values < 0.05 were considered to be statistically significant.
